# Social Cohesion, Social Participation, and HIV Related Risk among Female Sex Workers in Swaziland

**DOI:** 10.1371/journal.pone.0087527

**Published:** 2014-01-31

**Authors:** Virginia A. Fonner, Deanna Kerrigan, Zandile Mnisi, Sosthenes Ketende, Caitlin E. Kennedy, Stefan Baral

**Affiliations:** 1 Johns Hopkins Bloomberg School of Public Health, Department of International Health, Baltimore, Maryland, United States of America; 2 Johns Hopkins Bloomberg School of Public Health, Department of Health, Behavior, and Society, Baltimore, Maryland, United States of America; 3 Swaziland National AIDS Program, Mbabane, Swaziland; 4 Johns Hopkins Bloomberg School of Public Health, Department of Epidemiology, Baltimore, Maryland, United States of America; Institut Pluridisciplinaire Hubert Curien, France

## Abstract

Social capital is important to disadvantaged groups, such as sex workers, as a means of facilitating internal group-related mutual aid and support as well as access to broader social and material resources. Studies among sex workers have linked higher social capital with protective HIV-related behaviors; however, few studies have examined social capital among sex workers in sub-Saharan Africa. This cross-sectional study examined relationships between two key social capital constructs, social cohesion among sex workers and social participation of sex workers in the larger community, and HIV-related risk in Swaziland using respondent-driven sampling. Relationships between social cohesion, social participation, and HIV-related risk factors were assessed using logistic regression. HIV prevalence among the sample was 70.4% (223/317). Social cohesion was associated with consistent condom use in the past week (adjusted odds ratio [AOR]  = 2.25, 95% confidence interval [CI]: 1.30–3.90) and was associated with fewer reports of social discrimination, including denial of police protection. Social participation was associated with HIV testing (AOR = 2.39, 95% CI: 1.36–4.03) and using condoms with non-paying partners (AOR = 1.99, 95% CI: 1.13–3.51), and was inversely associated with reported verbal or physical harassment as a result of selling sex (AOR = 0.55, 95% CI: 0.33–0.91). Both social capital constructs were significantly associated with collective action, which involved participating in meetings to promote sex worker rights or attending HIV-related meetings/ talks with other sex workers. Social- and structural-level interventions focused on building social cohesion and social participation among sex workers could provide significant protection from HIV infection for female sex workers in Swaziland.

## Introduction

In sub-Saharan Africa, female sex workers have a 12-fold increase in odds of being HIV infected as compared to all women of reproductive age [1]. Sex workers face heightened behavioral risk for HIV infection due to high numbers of sex partners and frequent sexual encounters. This risk is exacerbated by social and structural factors, including stigma, poverty, sexual and physical violence [2]–[4], as well as inequitable laws and policies, police brutality, and lack of non-discriminatory healthcare services [5], [6].

In part due to the high HIV burden experienced by sex workers, recent mathematical models suggest that scaling up comprehensive community-based empowerment interventions among sex workers could avert significant numbers of new HIV infections not only among sex worker populations, but also among other reproductive-aged populations [7]. Evidence from HIV prevention interventions among sex workers in India [8]–[15], Brazil [16], [17], and the Dominican Republic [18] suggests that community empowerment can be an effective tool for reducing HIV-related risk and enacting social- and structural-level changes that alter HIV-related risk environments [19]. For example, the Avahan Initiative in India included interventions such as drop-in centers, peer education, sex-worker friendly health services, stakeholder advocacy, and formation of organizations led by and with sex workers to facilitate program ownership [20]. Avahan promoted social cohesion among sex workers, creating the potential for collective action, and worked to increase the acceptance of sex workers in the general community by facilitating social participation [21] through activities such as workshops bringing together police and sex workers [22].

**Figure 1:**
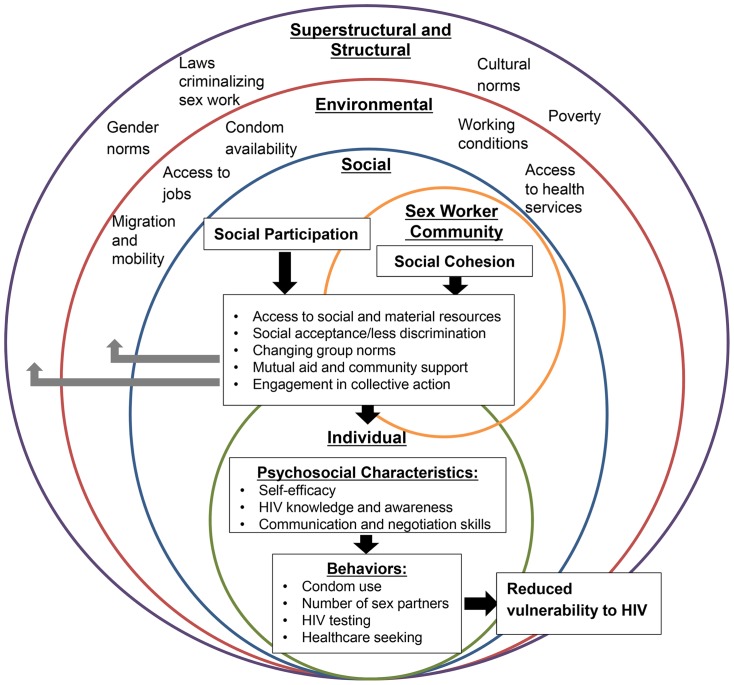
Theoretical framework of social capital and HIV-related risk among female sex workers in Swaziland.

Embedded within community empowerment models is the concept of social capital. Social capital concerns the connectedness of individuals between and within groups [23]. It encompasses the inherent value of social relationships and the material and social resources these relationships bring [23]. Robert Putnam, a prominent social capital theorist, has defined social capital as "the networks, norms, and social trust that facilitate co-operation for mutual benefit" [24]. According to Putnam, there are two main types of social capital: bonding, which refers to intra-group relationships, and bridging, which refers to inter-group interactions [24]. Although the current study involves two social capital constructs—social cohesion and social participation—we use the term social capital with caution as definitions vary and can be conceptually different [25]. In this study, social cohesion refers to mutual aid, trust, and solidarity present among sex workers and reflects bonding social capital. Social participation, which aligns with bridging social capital, refers to involvement in community groups outside of sex worker relationships, reflecting their inclusion in the larger society. A theoretical framework of social capital and HIV-related risk among sex workers is presented in Figure 1.

Extensive research has examined relationships between social capital and health [26]-[29], and social capital has been identified as a social-level factor influencing HIV vulnerability [30]. In general, public health outcomes tend to improve when social capital measures are high [31]. However, findings show these relationships are complex, especially in regards to HIV. For example, studies have found that HIV-associated risks can be positively or negatively associated with social capital, depending on the age and gender of the individual and the types of social ties maintained [32], [33]. Social capital may be especially important to disadvantaged groups, such as sex workers, because it increases access to material and social resources that would otherwise be denied to this socially excluded population [34]. However, little research has been conducted on social capital and HIV-related risk among female sex workers in sub-Saharan Africa [32].

HIV and Sex Work in Swaziland

Swaziland has the world’s highest HIV prevalence with an estimated 26% of adults aged 15–49 living with HIV [35]. The 2009 Modes of Transmission Study reports that heterosexual transmission accounts for the majority of HIV infections in Swaziland and sex work is a minor driver of the epidemic; however, it also acknowledges that little information is known about this hidden population [36]. A literature review revealed only two peer-reviewed studies conducted on sex workers in Swaziland [37], [38]. Although sex work is not criminalized in Swaziland, activities related to sex work, such as the solicitation and procurement of sex in public, are illegal [39], which makes conducting research among this population difficult yet essential to understanding factors shaping their HIV-related risk.

A situational analysis of sex work in Swaziland conducted in 2007 found that the majority of sex workers were young (below age 30) and most reported engaging in sex work due to poverty and lack of economic opportunities. Sex workers in Swaziland also reported experiencing high levels of violence, including violence perpetrated by clients and police, and lacked access to non-discriminatory health services, such as family planning services and testing for HIV and sexually transmitted infections [40]. Another study characterized both rural and urban sex work in Swaziland, highlighting that many sex workers are migratory and follow clients to “hot spots” depending on the season or time of month [41]. Sex workers in Swaziland are not brothel-based and obtain clients directly, either on the street or by being “on call” [41]. Street-based sex workers face heightened vulnerability to violence as the location of sex varies by client and includes isolated places where sex workers can be beaten, gang raped, or robbed [4]. Cohesion among street-based sex workers could help prevent violence and encourage behavior change, such as through collective agreements that no one will service a client without condoms. However, literature relating to the social context of sex work in Swaziland, including the social relationships that sex workers have with each other, is scarce. Given the potential importance of social factors, such as social cohesion and social participation, in reducing HIV-related risk and supporting community empowerment, this is a critically understudied area. Therefore, the aim of this study was to examine the relationship between social capital constructs, including social cohesion and social participation, and HIV-related risk among female sex workers in Swaziland.

## Methods

### Setting, study design, and participants

Women in Swaziland aged 18 years or older who reported exchanging sex for money, favors, or goods in the past twelve months were eligible to participate in the study. All participants provided informed consent prior to completing the questionnaire and serological testing. Oral consent scripts were used in lieu of signed consent forms to further ensure anonymity, protection of confidentiality, and security of participants. This ensured that in case consent forms were lost, no personal information or disclosure of sex work would take place. The study was implemented as a joint effort between the Swaziland government, Johns Hopkins University, and PSI Swaziland. Ethical approval for this study was granted by the Swaziland Scientific and Ethics Committee and the Johns Hopkins Bloomberg School of Public Health Institutional Review Board.

The sample size (n = 324) was calculated based on the ability to detect significant differences in HIV prevalence among participants with higher reported protective behaviors related to HIV, such as consistent condom use with paying partners (alpha = 0.05, power = 0.80).

Participants were recruited using respondent-driven sampling (RDS), which is a peer-driven chain referral sampling technique that approximates a random sample by mathematically controlling for inherent biases, including network size and homophily (the tendency of participants to recruit individuals who are similar to themselves) [42], [43]. To initiate the chain referral process, “seeds” were identified during formative research. Seeds were members of the sex worker community with diverse socio-demographic characteristics who were motivated to participate in the study and willing to recruit others in their social network. Three seeds were selected to begin the referral process and additional pre-selected seeds were brought into the study when accrual slowed. Each seed, and each subsequent participant who agreed to recruit other participants, was given referral cards with a 2-week expiration date to distribute to eligible members of their social network. Each recruiter could recruit a maximum of three participants. Upon survey completion, each participant was reimbursed for their time and travel to the study site, ranging from the equivalent of US$7-$23. Recruiters were also provided the equivalent of US$2.50 for every eligible participant who participated in the study. Recruiter-recruit relationships were tracked using linked card numbers.

### Data collection and survey measures

Data collection took place from July-November 2011. All participants completed a structured survey administered by a trained interviewer lasting approximately 1 hour in a private office setting. The survey could be completed in English or siSwati depending on the participant’s preference. Participants were asked questions relating to their social network (ascertained by asking how many different people a participant knew personally who were sex workers); socio-demographics; exposure to human rights violations; HIV-related knowledge, attitudes, and risk behaviors; condom negotiation skills; reproductive healthcare; and aspects of social capital, including social cohesion and social participation. Following completion of the survey, participants were tested for HIV by trained nurse counselors using rapid tests conducted on blood samples obtained via finger stick after providing informed consent. Unigold™ (Trinity Biotech) and Determine HIV 1® (Inverness Medical) were used as the preliminary screening and confirmatory HIV tests, respectively. Those who tested positive for HIV were given referrals to seek additional care and treatment through local health facilities.

### Social capital

Social cohesion was measured using a scale developed by Lippman, Kerrigan, and colleagues for use among sex worker populations in Brazil [17], [44]. Items comprising the scale were pilot-tested in Swaziland prior to survey implementation but required little change as questions asked about social situations, such as relying on colleagues for money or a ride to the hospital, that are applicable across populations. Participants were asked to rate their agreement or disagreement with statements relating to mutual aid, support, and trust among sex workers. Exploratory factor analysis (EFA) using polychoric correlations was used to determine unidimensionality within the social cohesion scale, and Cronbach’s alpha was used to assess the internal consistency [45]. Two of the 11 items were removed from the scale due to exhibiting low correlations with the other items and low factor loadings (below 0.3) in EFA. The remaining 9 items exhibited acceptable internal consistency (α = 0.76) and are listed in Table 1. For analysis the items were summed to create a scale with scores ranging from 0 to 27; scores were dichotomized at the median (16) for ease of interpretation, with 0–16 signaling low social cohesion and 16.1–27 signaling high social cohesion. Dichotomizing social capital scales is a technique previously employed by researchers examining associations between HIV and social capital [32].

**Table 1:** Measurement of social capital constructs and outcome variables.

	Items in scale/index	Properties	
		Mean (sd)	Alpha
Exposure Variables			
**Social Cohesion (scale)**		16.0 (5.2)	0.76
	You can count on your sex worker colleagues if you need to borrow money.		
	You can count on your sex worker colleagues to accompany you to the doctor or hospital.		
	You can count on your sex worker colleagues if you need to talk about your problems.		
	You can count on your sex worker colleagues if you need somewhere to stay.		
	You can count on your sex worker colleagues to help deal with violent or difficult client.		
	You can count on your sex worker colleagues to help you find other clients.		
	You can count on your sex worker colleagues to support the use of condoms.		
	The group of sex workers with whom you work is an integrated group.		
	You can trust the majority of other sex workers working in your area.		
	*(Response Choices: strongly agree, mostly agree, mostly disagree, or strongly disagree)*		
**Social Participation (index)**		1.4 (1.1)	n/a
	Participation in the following groups:		
	1. Affiliations with church or religious groups		
	2. Affiliations with clubs		
	3. Cultural activities		
	4. Community activities		
	*Participation was coded as “0” for no involvement and “1” for any involvement (being a member, active member, or leader of a specific social group)*		
**Outcome Variables**			
**Consistent condom use**	Consistency defined by having the number of reported protected sex acts (condom worn for entire duration of sex) equal to the number of all sex acts in the past week for all sex partners (vaginal or anal sex)		
**“Always” condom use**	Reported separately for: a) new clients, b) regular clients, and c) non-paying partners during vaginal sex. “Always” condom use vs. condom use reported never, rarely, sometimes, most of the time, or “don’t know”		
**HIV testing**	Have you been tested for HIV in the last 12 months?		
**HIV prevalence**	HIV testing was offered to all study participants during data collection		
**Social discrimination**	Have you ever felt rejected by your friends as a result of you selling sex?		
	Have you ever felt afraid to seek healthcare services as a result of you selling sex?Have you ever felt that the police refused protection as a result of you selling sex?		
	Have you ever felt any verbal and physical harassment as a result of you selling sex?		
	Have you ever been beaten up as a result of you selling sex?		
**Collective action**	In the past 12 months, have you participated in: 1) A meeting, march, rally, or gathering to promote the rights of sex workers? Or 2) Any talks or meetings related to HIV/AIDS with other sex workers?		

Social participation was measured by asking participants about their involvement in groups outside their sex worker network, including church or religious groups, clubs (e.g., sports, student groups, women’s clubs), cultural activities (e.g., dance, music), and community activities. Participants were scored based on the intensity of their involvement (0 =  do not participate, 1 = member, 2 = active member, and 3 = group leader). This assessment is similar to the method for measuring structural social capital in the World Bank’s Social Capital Tool [46], which has also been used to measure social participation among sex worker populations in Brazil [17], [44]. Categories of social participation were adapted to the Swazi context prior to implementation. Due to the skewed distribution of data for this construct (mean score = 1.6 on a 16-point scale), a social participation index was generated by summing participants’ involvement across the various groups (“0” for no involvement and “1” for any involvement) for analysis. Scores on the social participation index could range from 0 (no social participation) to 4 (participation in all types of social involvement). The median score on the social participation index was 1, and scores were dichotomized at the median (0 =  score of 0 or 1; 1 =  score >1) for ease of interpretation and due to the skewed distribution of the data.

Collective action, which is defined as any action aiming to improve a group’s condition [47], has been described as both an output measure of social capital and an important proxy for assessing social capital itself [48]. In this study, collective action was treated as an outcome variable as it was hypothesized that having high social cohesion and/or social participation could lead to collective action, including engagement in activities related to HIV prevention. Collective action was measured through two questions asking about participation within the past 12 months in: 1) meetings, marches, rallies, or gatherings to promote the rights of sex workers, or 2) talks or meetings related to HIV/AIDS with other sex workers.

### HIV-related risk

Behavioral HIV-related risk was measured by asking participants about condom use during vaginal and anal sex with different partner types, including new clients, regular clients, and non-paying partners. New clients were defined as paying clients with whom participants had had sex once or twice. Regular clients were defined as those with whom participants had had sex at least three times and who pay for sex. Non-paying partners included sexual partners of participants, such as husbands and boyfriends, who do not pay for sex.

For purposes of this study, consistent condom use was defined as reporting condom use in all sex acts in the past week with all partners, which was measured by constructing a ratio of the reported number of protected sex acts to all sex acts reported in the previous week. “Always” condom use in the past 30 days versus some or no condom use was also measured for new clients, regular clients, and non-paying partners. Although this study measured “always” condom use in both anal and vaginal sex, results in this analysis were restricted to condom use during vaginal sex as approximately 65% of the study population reported no engagement in anal sex. Prior HIV testing was measured by asking participants whether they had received an HIV test in the past 12 months.

Additionally, several factors relating to violence and social discrimination were included as outcome variables since previous research has shown that violence, including physical, sexual, and emotional violence, is associated with elevated HIV risk, especially among female sex workers [49], [50]. Social discrimination outcomes included reported rejection by friends, reported refusal of protection from police, feeling afraid to seek health services, reported verbal or physical abuse, and having been beaten up due to sex work. All exposure and outcome variables are defined in Table 1.

### Analysis

All analyses were conducted using Stata version 11. Cross-sectional data were used to assess the relationship between HIV-related outcomes and social capital constructs, including social cohesion and social participation. Chi square tests were performed to assess differences in the dichotomized social cohesion and social participation scores across socio-demographic characteristics.

To account for biases inherent in RDS data collection, including recruiting patterns and network size, data were weighted based on population estimates of the outcome variable in all analyses. Univariate logistic regression analyses were performed on HIV-related outcome variables to assess associations with social cohesion and social participation. For analyses where either social cohesion or social participation were associated with the outcome at p≤0.1, multivariate regressions were conducted to assess the impact of the relationship when controlling for age, income, education, marital status, and geographic region of residence in addition to the RDS weights, which were identified *a priori* as potential confounders between social capital and HIV-related variables.

However, there is no consensus in the field about how RDS data should be treated in multivariate analysis [51], particularly because traditional weighing methods can produce incorrect standard errors [52]. Therefore, sensitivity analyses were performed in which data were left completely unadjusted and then adjusted using network size as a confounding variable, which is a technique previously used for conducting multivariate logistic regression analyses with RDS data [53]. As the sensitivity analyses showed no significant difference in associations, analyses using weighted RDS data were employed for all subsequent analyses. Additionally, analyses were performed with and without “seed” data because seeds were non-randomly selected. As no significant differences in associations were detected, data from seeds remained in the analysis.

## Results

### Study population

Data were collected from 325 female sex workers, including data from nine non-randomly selected seeds. The median age of participants was 25 (inter-quartile range [IQR] 21–30). About half of participants (n = 175) had completed some secondary or high school. Out of 317 participants with a confirmed HIV test result, 70.6% (223/317) tested HIV positive. Over 54% of participants (176/322) reported being told they were HIV positive by a healthcare provider sometime before participating in the study. Most participants were single and had never been married (285/321). Table 2 contains socio-demographic characteristics of the sample. Marital status was associated with social cohesion in that participants who had ever cohabitated with a sexual partner (e.g., married, widowed, or divorced) experienced lower levels of social cohesion compared to participants who were single/never married (p = 0.05).

**Table 2:** Socio-demographic characteristics of the sample by levels of social cohesion and social participation.

		Social Cohesion			Social Participation		
**Demographic characteristic**	**N (%)**	**High N**	**Low N**		**High N**	**Low N**	
**Study Populati**o**n** (total)	325	142	159				
**Age** (in years)				0.96			0.43
<21	64 (19.7)	30	30		31	30	
21–24	82 (25.2)	35	39		37	45	
25–29	91 (28.0)	39	47		45	44	
30+	88 (27.1)	38	43		34	52	
**Education**				0.89			0.22
Primary or lower	106 (32.6)	45	51		44	60	
Some secondary	175 (53.9)	79	85		87	85	
Completed secondary or higher	44 (13.5)	18	23		16	26	
**Marital Status**				0.05			0.10
Ever cohabited	36 (11.2)	11	24		12	24	
Single/Never married	285 (88.8)	129	135		133	145	
**Region of residence**				0.68			0.19
Hhohho	102 (31.4)	40	53		49	48	
Manzini	159 (48.9)	71	77		64	95	
Shiwelweni	57 (17.5)	28	25		31	25	
Lubombo	6 (1.8)	3	3		2	3	
**Income (in Emalangeni)**				0.09			0.08
0–450	85 (26.2)	32	46		43	39	
451–800	93 (28.6)	45	38		46	46	
801–1300	63 (19.4)	33	26		30	32	
1300+	84 (25.8)	32	49		28	54	
**HIV status**				0.56			0.60
Uninfected	94 (29.7)	30	49		45	47	
Infected	223 (70.3)	99	107		100	119	

### Social capital among sex workers

Social cohesion scores among sex workers were relatively high. On a 27 point scale, the mean and median score was 16 (range 0–27). Over 60% of sex workers agreed or strongly agreed they could turn to sex workers with whom they work if they needed money, a ride to the hospital, someone to discuss their problems with, or help with a difficult client. However, only 37% of participants (120/321) agreed with statement, “You can trust the majority of other sex workers working in your area.”

Social participation was generally low. On a four point scale, the median score was 1 (range 0–4). No social participation was reported by 21% of participants (67/318). Affiliation with a church or religious group was the most commonly reported form of social participation with 61% reporting such involvement (197/323).

Regarding collective action, 54% (172/320) reported attending a talk or meeting relating to HIV with other sex workers in the past 12 months, but only 34% (111/324) reported attending a meeting, march, rally, or gathering in the past 12 months to promote the rights of sex workers.

### Associations between social cohesion and HIV-related risk

Table 3 presents results from logistic regression analyses examining social cohesion and HIV-related behaviors and risk factors. In univariate and multivariate analysis, social cohesion was significantly associated with consistent condom use in the past week with all partners (adjusted odds ratio [AOR]  =  2.25, 95% confidence interval [CI]: 1.30–3.90). However, there were no associations found between social cohesion and “always” condom use with new partners, regular partners, or non-paying partners. Social cohesion was also associated with fewer reports of social discrimination, including reported refusal of police protection (AOR = 0.53, 95% CI: 0.31–0.90) and reported rejection by friends due to sex work (AOR = 0.52, 95% CI: 0.32–0.84). Social cohesion also trended towards being inversely associated with feeling afraid to seek services due to selling sex (AOR = 0.67, 95% CI: 0.41–1.08, p = 0.10), but results were not significant. Having high levels of social cohesion was significantly associated with one measure of collective action: participating in meetings to promote sex worker rights (AOR = 2.33, 95% CI: 1.37–3.94).

**Table 3:** Associations of social cohesion and HIV-related outcomes.

		Social Cohesion n(%)		Unadjusted^a^		Adjusted^b^	
Outcome variable	n	High	Low	OR (95% CI)	P value	AOR (95% CI)	P value
**Behavioral**							
Consistent condom use- all partners	278	99/135 (73.3)	80/143 (55.9)	2.17 (1.30–3.60)	0.003	2.25 (1.30–3.90)	0.004
“Always” condom use- new clients	280	101/133 (75.9)	106/147 (72.1)	1.22 (0.71–2.09)	0.47	-----	
“Always” condom use- regular clients	291	68/138 (49.3)	70/153 (45.8)	1.15 (0.72–1.83)	0.55	-----	
“Always” condom use- non-paying partners	267	42/127 (33.1)	44/140 (31.4)	1.08 (0.64–1.81)	0.76	-----	
Tested for HIV in previous year	300	108/142 (76.1)	114/158 (72.2)	1.23 (0.73–2.07)	0.44	-----	
**Biological**							
HIV-infected	294	99/138 (71.7)	107/156 (68.6)	1.16 (0.70–1.93)	0.56	-----	
**Social Discrimination/Violence**							
Afraid to seek health services	301	56/142 (34.4)	78/159 (49.1)	0.67 (0.43–1.07)	0.10	0.67 (0.41–1.08)	0.10
Felt rejected by friends	318	63/142 (44.4)	97/159 (61.0)	0.51 (0.32–0.81)	0.004	0.52 (0.32–0.84)	0.008
Was refused police protection	300	59/141 (41.8)	91/159 (57.2)	0.54 (0.34–0.85)	0.009	0.53 (0.31–0.90)	0.02
Verbal/physical harassment	301	84/142 (59.2)	102/159 (64.2)	0.81 (0.51–1.29)	0.36	-----	
Beaten up due to selling sex	299	49/140 (35.0)	65/159 (26.4)	0.78 (0.49–1.25)	0.30	-----	
**Collective Action**							
Participated in meeting to promote sex worker rights	301	59/142 (41.6)	42/159 (26.4)	1.98 (1.22–3.23)	0.006	2.33 (1.37–3.94)	0.006
Participated in meeting about HIV/AIDSwith other sex workers	314	87/142 (61.3)	77/157 (49.0)	1.64 (1.03–2.61)	0.04	1.61 (0.96–2.68)	0.07

a Univariate analyses adjusted for RDS weights based on estimated population proportions of outcome variable.

b Adjusted for age, income, education, marital status, and region in addition to RDS weights.

### Associations between social participation and HIV-related risk

Associations between social participation and HIV-related behaviors and risk factors are presented in Table 4. In both univariate and multivariate regressions, being tested for HIV in the previous 12 months was significantly associated with having high levels of social participation (AOR = 2.39, 95% CI: 1.36–4.02). When the analysis excluded HIV-infected participants who did not test for HIV in the past 12 months but had previously been told by a healthcare provider that they were HIV infected (n = 27), the association between social participation and prior testing remained significant (AOR = 1.99, 95% 1.02–3.86, p = 0.04). Additionally, reporting “always” condom use with non-paying partners was associated with social participation (AOR = 1.99, 95% CI = 1.13–3.51), although social participation was not significantly associated with any other condom use measure. Participants with high levels of social participation had a 45% reduction in odds of reporting experiencing verbal or physical harassment as a result of selling sex compared to those with low levels of participation (AOR = 0.55, 95% CI: 0.33–0.91). Social participation, like social cohesion, was significantly associated with collective action. Compared to participants with low levels of social participation, participants with high levels of social participation had over twice the odds of participating in meetings to promote sex worker rights (AOR = 2.26, 95% CI: 1.35–3.78).

**Table 4:** Associations of social participation and HIV-related outcomes.

		Social Participation n(%)		Unadjusted^a^		Adjusted^b^		
Outcome variable	n	High	Low	OR (95% CI)	P value	AOR (95% CI)	P value	
**Behavioral**								
Consistent condom use- all partners	294	86/135 (63.7)	103/159 (64.8)	0.95 (0.60–1.54)	0.85	-----		
“Always” condom use- new clients	294	101/133 (75.94)	117/161 (72.7)	1.19 (0.70–2.02)	0.53	-----		
“Always” condom use- regular clients	306	70/142 (49.3)	78/164 (47.6)	1.07 (0.68–1.68)	0.76	-----		
“Always” condom use- non-paying partners	279	53/132 (40.2)	41/147 (27.9)	1.73 (1.05–2.87)	0.03	1.99 (1.13–3.51)		0.04
Tested for HIV in previous year	317	121/147 (82.3)	117/170 (68.8)	2.11 (1.23–3.61)	0.007	2.39 (1.36–4.02)		0.003
**Biological**								
HIV-infected	311	100/145 (70.0)	119/166 (71.7)	0.88 (0.54–1.43)	0.60	-----		
**Social Discrimination/Violence**								
Afraid to seek health services	318	63/147 (42.9)	78/171 (45.6)	0.89 (0.57–1.40)	0.62	-----		
Felt rejected by friends	318	78/147 (53.1)	89/171 (52.1)	1.04 (0.67–1.62)	0.86	-----		
Was refused police protection	317	67/146 (45.9)	91/171 (53.2)	0.75 (0.48–1.16)	0.20	-----		
Verbal/physical harassment	318	79/147 (53.7)	115/171 (67.2)	0.57 (0.36–0.89)	0.02	0.55 (0.33–0.91)		0.02
Beaten up due to selling sex	315	51/146 (34.9)	73/169 (43.2)	0.71 (0.45–1.12)	0.14	-----		
**Collective Action**								
Participated in meeting to promote sex worker rights	318	64/147 (43.5)	46/171 (26.9)	2.09 (1.31–3.36)	0.002	2.26 (1.35–3.78)		0.002
Participated in meeting about HIV/AIDS with other sex workers	314	85/145 (58.6)	83/169 (49.1)	1.47 (0.94–2.30)	0.09	1.31 (0.79–2.17)		0.30

a Univariate analyses adjusted for RDS weights based on estimated population proportions of outcome variable.

b Adjusted for age, income, education, marital status, and region in addition to RDS weights.

## Discussion

Given the HIV prevalence among this sample of female sex workers was almost three times the general population prevalence in Swaziland, developing HIV prevention activities for this population is critical. Associations between social capital constructs and HIV-related risk factors found in this study demonstrate that including social capital enhancement in HIV prevention interventions for sex workers in Swaziland could be beneficial. For example, we found that having high levels of social cohesion was associated with increased consistent condom use among sex workers and their sexual partners and was negatively associated with certain aspects of social discrimination. Having high levels of social participation was associated with consistent condom use between sex workers and their non-paying partners and with being tested for HIV in the previous year, and was associated with fewer reports of verbal or physical harassment. Both social cohesion and social participation were associated with participating in collective action with other sex workers.

Interestingly, on the social cohesion scale, reported trust among sex workers in a particular area was relatively low while feelings of mutual aid were generally high. This difference could be a consequence of question wording (as the “trust” question asked about “sex workers working in your area” and the “mutual aid” questions asked about “sex workers with whom you work”), but it could also provide insight into the nature of sex work in Swaziland. Research among sex workers in South Africa similarly found limited trust between sex workers due to circumstances such as competition for clients, but acts of support, such as visiting each other in the hospital or borrowing money, were common [54]. Other studies among sex workers have found that building cohesion can be challenging not only due to lack of trust but also due to heterogeneity between sex workers[55], lack of common identity [56], frequent migration and high turn-over rates [57],and not wanting to be openly identify as a sex worker [55]. Despite these challenges, interventions aiming to improve cohesion found that over time, cohesion was built by allowing sex workers the space and time to come together to share problems and develop collective solutions [56] and by having sex workers advise on intervention development and implementation to develop a sense of community and ownership [21], [58].

The association between high levels of social cohesion and consistent condom use found in this study is consistent with previous research that has examined these variables longitudinally. The Sonagachi Project from Kolkata, India, which sought to reduce HIV risk for sex workers through promoting group solidarity, fostering empowerment, and increasing access to social and material resources, has led to significant increases in condom use among sex workers [10]. However, social cohesion was not directly measured in the Sonagachi Project. A replication of this intervention in West Bengal also led to increases in consistent condom use [59], as have several other interventions focusing on community mobilization and collectivization of sex workers in India [9], [60] and on community solidarity in the Dominican Republic [18] and Brazil [17]. The negative association of social cohesion and refusal of police protection found in this study is consistent with results from interventions in India, such as the Avahan initiative, which demonstrated that groups of sex workers can collectively confront and change police practices towards sex workers, resulting in safer and healthier work environments [22], [61].

The association between social participation and “always” condom use with non-paying partners is noteworthy as condom use among sex workers has been found to be lower in partnerships with high relationship intimacy and trust [62]. Having sexual relationships with non-paying partners is linked to social participation in that both involve making connections with the larger community beyond the world of sex work, which could explain why social participation was related to condom use with non-paying partners but not with new or regular clients in this study. The significant association between social participation and previous HIV testing has not been reported in other studies with sex workers, but research with other high risk populations has found that acceptability of HIV testing was associated with having prosocial network characteristics [63], and that having a small social network was associated with not being tested for HIV [64]. As global testing rates remain low, further research about the influence of social networks and social participation on HIV testing could generate important insights for increasing uptake of HIV testing, particularly among population groups at heightened risk for HIV infection.

Other research has demonstrated that the type of social participation influences the relationship to HIV risk. For example, a study from South Africa found that participation in savings clubs was associated with risky sexual behavior among female members and with being HIV infected among male members [33]. Prior research has shown that the value of participating in certain social groups is dependent on characteristics of the group, such as how well the group functions, relationships among group members, and the group’s purpose, which could all contribute to how protective or harmful group membership is in relation to HIV [65]. In this study, social participation was examined collectively, but in further research it would be beneficial to look at associations by participation in different group types and their function. The IMAGE study, which involved microfinance and group health education components for women in South Africa, provides an innovative model for interventions striving to improve economic opportunities for disadvantaged groups while simultaneously creating channels for social participation and fostering social cohesion [32].

### Limitations

This study has several limitations. Firstly, since this is a cross-sectional study, causation and temporality between exposure and outcomes cannot be determined. Additionally, this study utilized respondent-driven sampling methods. While RDS has led to important breakthroughs for sampling hard to reach populations, questions remain about biases introduced by purposeful seed selection and preferential referral patterns of subsequent recruits [66]. There is also a lack of consensus about how to treat RDS data in multivariate regression analysis [51]. It is possible that using RDS led to a more homogeneous population of sex workers than currently exists in Swaziland. For example, previous research suggests that transactional sex is common in rural areas of Swaziland, but that the nature of the exchange is based more on “neighborliness” (i.e., helping out a neighbor in need of money or food by exchanging sex) than sex work [41]. This study focused more on “open” sex workers operating in urban and peri-urban areas.

Most questions analyzed in this study came from self-reported behaviors, such as condom use, which introduces social desirability and reporting bias. Additionally, this study measured complex social capital constructs with a social cohesion scale and a social participation index that have not undergone extensive reliability and validity testing and may not be applicable to other populations. Social capital is a social phenomenon and is therefore a characteristic of a group and not just a single individual [67]; however, in this study social capital was measured on an individual basis. Although this is a common way to measure social capital, there are debates in the field about the utility of using individual measurement as a proxy for this ecological concept [68]. Additionally this study focused on social participation and social cohesion as exogenous exposure variables and did not explore the personal and environmental factors that may facilitate or hinder an individual’s propensity for social engagement.

## Conclusion

Few studies have examined the social context of sex work in sub-Saharan Africa, and we identified only two HIV prevention interventions targeting sex workers in this region that sought to address social and structural factors related to HIV [69], [70]. Most research on social capital- and community empowerment-based interventions for sex workers has been conducted in areas of the world, such as South Asia, where sex work is largely establishment-based. This feature may facilitate interventions aimed at increasing social cohesion and participation as establishments may be easy ways to bring together sex workers into functioning groups. Sex work in sub-Saharan Africa often involves non-establishment based sex work [71], presenting an additional challenge for implementing HIV prevention interventions for sex workers in these settings.

Results from this study demonstrate that the social context of sex work matters. Findings suggest that higher levels of social cohesion and social participation are associated with protective behaviors, including condom use and HIV testing, and are inversely associated with HIV-related risk factors, such as experiencing social discrimination and violence. However, more needs to be understood about the social context of female sex workers in Swaziland, such as the extent to which sex workers are willing and able to collectivize and advocate for rights and how sex workers are portrayed and treated by the larger community. Answering these questions is critical to understanding how community-based empowerment interventions, which could include components related to building social capital among sex workers and between sex workers and the larger community, can be implemented in this setting. Given recent evidence that scaling-up comprehensive community-based empowerment interventions is a cost-effective way to prevent a substantial number of new infections, especially in places with high rates of HIV infection [7], a better understanding of these relationships is important not only for the sex worker community, but also for the general population.
